# Supplementary material for: Venomix: a simple bioinformatic pipeline for identifying and characterizing toxin gene candidates from transcriptomic data
Source: PeerJ. 2018 Jul 31;6:e5361. doi: 10.7717/peerj.5361 (PMC6074769; doi:10.7717/peerj.5361)
Supplement: Supplemental Information 3 [file peerj-06-5361-s003.gz › FinalOutput_E-6/Neutral_phospholipase_A2_3_21/finaltree.pdf]

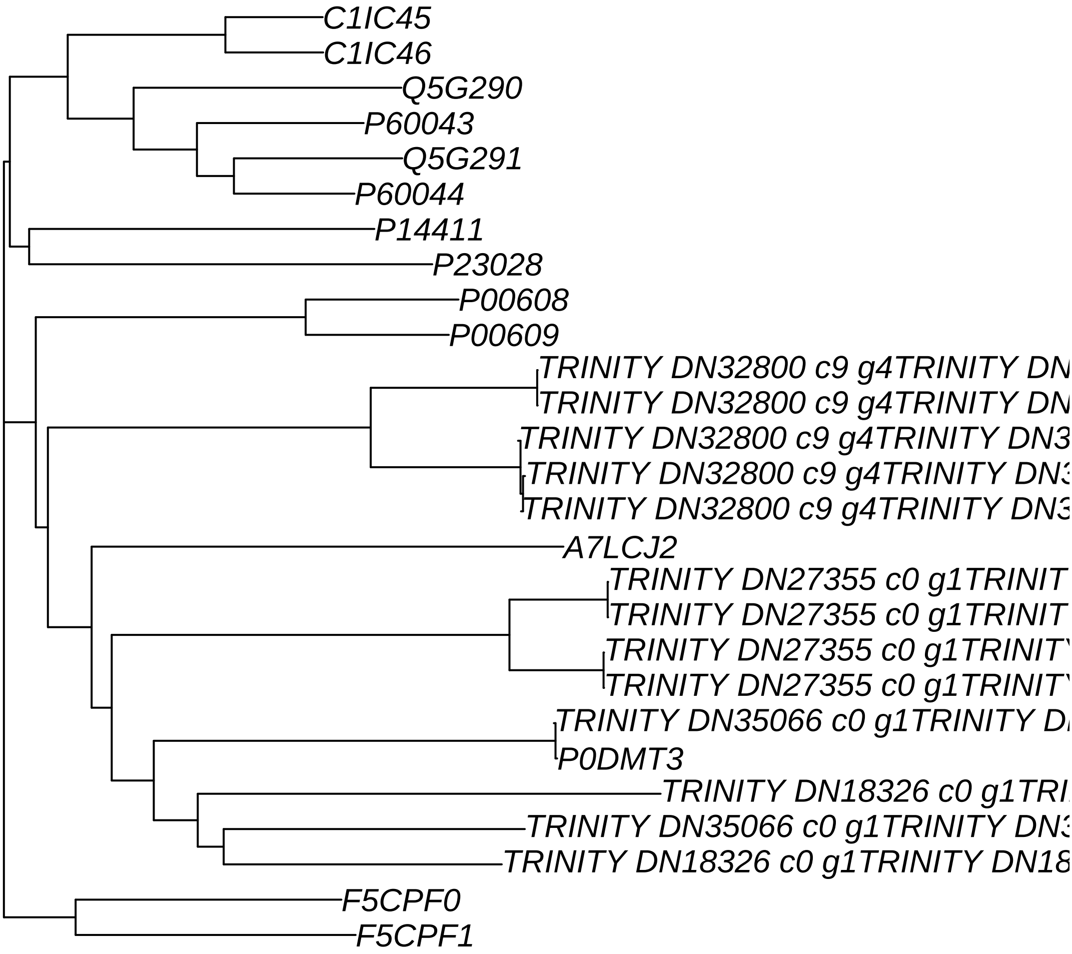
C1IC45
C1IC46
Q5G290
P60043
Q5G291
P60044
P14411
P23028
P00608
P00609
TRINITY DN32800 c9 g4TRINITY DN
TRINITY DN32800 c9 g4TRINITY DN
TRINITY DN32800 c9 g4TRINITY DN3
TRINITY DN32800 c9 g4TRINITY DN3
TRINITY DN32800 c9 g4TRINITY DN3
A7LCJ2
TRINITY DN27355 c0 g1TRINIT
TRINITY DN27355 c0 g1TRINIT
TRINITY DN27355 c0 g1TRINITY
TRINITY DN27355 c0 g1TRINITY
TRINITY DN35066 c0 g1TRINITY D
P0DMT3
TRINITY DN18326 c0 g1TRI
TRINITY DN35066 c0 g1TRINITY DN3
TRINITY DN18326 c0 g1TRINITY DN18
F5CPF0
F5CPF1
